# Response to a prehabilitation program for patients with oesophageal cancer: an observational study

**DOI:** 10.1186/s13741-025-00633-6

**Published:** 2025-12-19

**Authors:** Elja A. E. Reijneveld, Jaap J. Dronkers, Sandra Beijer, Miranda J. Velthuis, Ad Kerst, Stefan Bos, Tamara Warmelink-Galema, Jelle P. Ruurda, C. Veenhof, ‘PRIOR study group’, Elles Steenhagen, Femke van Leeuwen, Richard van Hillegersberg, Jan Willem Haveman, Joyce M.A. Stel, Dolf Liest, Bea Sijtema, Ewout A. Kouwenhoven, Iris Mekenkamp, Margreet Tinselboer, Corine van Dijk, Miron Sauer, Peter van Duijvendijk, Joran Kauw, Willeke Frank, Anthoinet Freriksen, Norma Schokker, Edwin J. van Adrichem

**Affiliations:** 1 Research Center for Healthy and Sustainable Living, Research Group Innovation of Human Movement Care, HU University of Applied Sciences Utrecht, Heidelberglaan 7, Utrecht, 3584 CS the Netherlands; 2 Department of Dietetics, Maastricht University Medical Center+, P. Debyelaan 25, Maastricht, 6229 HX The Netherlands; 3 Netherlands Comprehensive Cancer Organisation (IKNL), Rijnkade 5, Utrecht, 3511 LC The Netherlands; 4 Department of Rehabilitation, Physiotherapy Science and Sport, Brain Center, University Medical Center Utrecht, Heidelberglaan 100, Utrecht, 3584 CX The Netherlands; 5 Department of Dietetics, University Medical Center Groningen, Hanzeplein 1, Groningen, 9713 GZ The Netherlands; 6 Department of Physiotherapy, Isala Hospital, Dokter Van Heesweg 2, Zwolle, 8025 AB The Netherlands; 7 Department of Surgery, University Medical Centre Utrecht, Heidelberglaan 100, Utrecht, 3584 CX The Netherlands

**Keywords:** Preoperative Care, Prehabilitation, Oncology, Physical fitness, Nutritional status, Oesophageal Cancer

## Abstract

**Background:**

To optimize prehabilitation for patients with oesophageal cancer, insight is required into the response to prehabilitation, and factors affecting this response. This study investigated (1) the response to prehabilitation in patients with oesophageal cancer following neoadjuvant treatment, (2) the association between baseline physical fitness and preoperative changes in fitness, (3) differences in physical fitness, nutritional status, and fatigue between responders and non-responders to prehabilitation.

**Methods:**

This multicenter cohort study included patients with oesophageal cancer, following a 6–10 week personalized prehabilitation program as part of standard care. Prehabilitation, consisting of supervised exercise and nutritional counseling, started after neoadjuvant treatment. Preoperative physical fitness and nutritional status were monitored before and after neoadjuvant treatment, and after prehabilitation. Changes over time were analyzed using linear mixed models. Impact of baseline fitness on preoperative changes in exercise capacity was investigated using a linear mixed regression model. Differences between responders to prehabilitation (> 0 Watt improvement during exercise training) and non-responders were analyzed using Independent T-Tests and multivariable logistic regression.

**Results:**

Two hundred forty patients were included (mean age 66.0 (9.3) years; 77.1% male). On average, physical fitness and nutritional status declined during neoadjuvant treatment, and significantly improved during prehabilitation. Exercise capacity increased by + 32.7 Watts (95% CI: 25.2–40.2) during prehabilitation, with similar improvements across patients with low, moderate, and high baseline capacity. Substantial heterogeneity in preoperative changes was observed, with only 49.6% of patients following a pattern of decline-improvement (corresponding to average values for exercise capacity). Sixty-five percent of patients were classified as responders. Greater decline in fitness during neoadjuvant treatment (*p* < .001), lower fitness after neoadjuvant treatment (*p* = .001), and higher fatigue (*p* = .01) were associated with a positive response to prehabilitation.

**Conclusions:**

On average, patients with oesophageal cancer improved in physical fitness and nutritional status during prehabilitation, recovering from the impact of neoadjuvant treatment. Response to prehabilitation was independent of baseline fitness. A greater decline in fitness during neoadjuvant treatment, lower fitness before prehabilitation and higher fatigue were associated with a positive response. The heterogeneity in preoperative trajectories among patients underscores the importance of regular monitoring to tailor interventions to individual needs.

**Supplementary Information:**

The online version contains supplementary material available at 10.1186/s13741-025-00633-6.

## Introduction

The standard curative treatment for patients with oesophageal cancer consists of neoadjuvant chemoradiotherapy followed by oesophagectomy, which is associated with a high risk of postoperative complications, as well as a decline in quality of life, nutritional status, and physical fitness of patients (Grotenhuis et al. 2010; Blencowe et al. 2012; Reijneveld et al. 2022a; Gannon et al. 2017; Wilk et al. 2023). The risk of postoperative complications and morbidity appears to increase when preoperative physical fitness levels and nutritional status are poor, or have deteriorated during neoadjuvant treatment (Park et al. 2023; Motoori et al. 2018; Sheill et al. 2020; Bor et al. 2021). Consequently, prehabilitation programs are increasingly being integrated into standard care pathways to enhance postoperative recovery (Guinan et al. 2016; Doganay and Moorthy 2019; Bausys et al. 2022).

Prehabilitation refers to the preoperative optimization of physical fitness before major surgery and may be unimodal (e.g. exercise only) or multimodal (including exercise, nutrition, psychological, and/or lifestyle interventions) (Silver and Baima 2013; Bolshinsky et al. 2018). In patients with oesophageal cancer, these programs may be initiated after completion of neoadjuvant treatment, during the 6–10 week rest period before surgery, or may begin during neoadjuvant treatment. Prehabilitation programs are promising in improving preoperative physical fitness and nutritional status, reducing the risk of postoperative complications, and enhancing postoperative recovery in patients with oesophageal cancer (Halliday et al. 2021a, 2021b; Janssen et al. 2022; Minnella et al. 2018).

Previous research indicates that physical fitness and nutritional status generally decline during a 5-week period of neoadjuvant treatment, and improve during a 6–10 week prehabilitation period before surgery (Halliday et al. 2021b; Janssen et al. 2022; Argudo et al. 2021). However, substantial heterogeneity exists in preoperative trajectories across individual patients (Halliday et al. 2021b; Harada et al. 2025; Reijneveld et al. 2025). In addition, further insight is required into factors associated with the response to preoperative exercise programs (Bor et al. 2021; Halliday et al. 2021b). This knowledge is essential to identify which patients benefit most from prehabilitation, and to optimize and personalize program content, as recommended in recent studies (Bausys et al. 2022; Tukanova et al. 2022; Scheede-Bergdahl et al. 2019; Gillis et al. 2021). Previous research suggests that patients with lower fitness levels prior to starting exercise prehabilitation are more likely to show improvement (Halliday et al. 2021b; Minnella et al. 2016; Cate et al. 2024). However, it is unclear whether a decline in physical fitness during neoadjuvant treatment is related to subsequent changes in fitness during the exercise training period prior to surgery. Baseline fitness at the start of neoadjuvant treatment may also influence the preoperative trajectory of fitness, but this relationship has been largely unexplored (Harada et al. 2025). Additionally, common symptoms such as fatigue and malnutrition may affect the response to prehabilitation, yet their impact on exercise training outcomes remains insufficiently studied and poorly understood (Gillis et al. 2021, 2022; Ferreira et al. 2021; Reijneveld et al. 2024). Understanding the dynamic changes in preoperative physical fitness- and the factors that influence them- is essential for enabling timely and targeted interventions.

Therefore, this study aimed to (1) describe the average response to a prehabilitation program in patients with oesophageal cancer following neoadjuvant treatment, and to explore the heterogeneity in individual responses, (2) examine whether baseline physical fitness is associated with preoperative changes in physical fitness, and (3) explore whether characteristics related to physical fitness, nutritional status, and fatigue differ between responders and non-responders to prehabilitation.

## Methods

### Study design and participants

The PRIOR (PReoperative intervention to Improve outcomes in patients with Oesophageal cancer after Resection) study is a multicenter, observational cohort study evaluating the implementation of a prehabilitation program to improve physical fitness and nutritional status of patients with oesophageal cancer. All patients in the University Medical Center Utrecht, University Medical Center Groningen, Gelre Hospital, Isala Hospital, and Hospital Group Twente who were treated with curative intent, participated in the PRIOR program as part of the standard care pathway. Patients were enrolled in the study if they were scheduled to undergo curative treatment, consisting of neoadjuvant chemoradiotherapy followed by esophagectomy, at one of the participating centers. There were no exclusion criteria. After diagnosis and before the start of neoadjuvant treatment, patients were informed about the study and signed informed consent for the use of their (anonymized) data for scientific research. Patients were included in the period between March 2018 and January 2020. The study protocol was approved by the Medical Ethics Committee of the University Medical Centre Utrecht (protocol number 17–533/C). The study was reported according to the STrengthening the Reporting of OBservational studies in Epidemiology (STROBE) statement for cohort studies (Elm et al. 2008).

### PRIOR prehabilitation program

The prehabilitation program involved two periods, consisting of a neoadjuvant treatment period, and an exercise training period.

#### Neoadjuvant treatment period

Neoadjuvant treatment started after diagnosis, with a 5-week schedule of chemoradiotherapy. During this period, patients received a general advice from a physiotherapist to engage in physical activity of moderate intensity – for example walking and cycling—for at least 150 min every week, spread over several days, in accordance with the Dutch physical activity guidelines (Weggemans et al. 2018). Furthermore, patients received nutritional support provided by a dietician, to maintain body weight and achieve an adequate protein and energy intake, according to the European Society for Clinical Nutrition and Metabolism (ESPEN) guidelines for patients with cancer and the Dutch dietary guidelines (Muscaritoli et al. 2021; Dutch malnutrition guideline 2024). Patients were regularly monitored by a dietician, and if the nutritional intake did not meet individual requirements, patients received oral nutritional supplements or enteral tube feeding.

#### Exercise program period

During the 6- to 10-week rest-period between neoadjuvant treatment and surgery, recommendations regarding moderate intensity physical activity (150 min/week), and nutritional support—aimed at maintaining body weight and ensuring adequate protein and energy intake—were sustained. In addition, patients followed an exercise training program supervised by a physiotherapist in primary care practice to improve muscle function and aerobic capacity. Physiotherapists were provided with a guideline detailing the content of the exercise program, including training frequency and intensity. The guideline recommended three exercise sessions per week (two supervised and one home-based), incorporating both resistance and aerobic components. Resistance exercises were prescribed in three sets of 12 repetitions, targeting an exertion level of 4–5 on the Borg Rating of Perceived Exertion Scale (Borg score 0–10) (Borg 1998). Aerobic exercise was advised to be performed at a mean intensity corresponding to 40 to 50% of peak workload achieved during the Steep Ramp Test (de Backer et al., 2007; Royal Dutch Society for Physical Therapy and the Association of Cesar and Mensendieck 2022). Physiotherapists were permitted to tailor the exercise program to individual patients, taking into account their motivation and physical capacity.

### Measurements

#### Patient characteristics

Patient characteristics, including age, gender, body mass index (BMI), tumor type and stage, medical treatment regimen, American Society of Anesthesiologists (ASA) classification (assessing the overall physical health status of participants prior to surgery) (American Society of Anesthesiologists Classification (American n.d.)) and comorbidity were registered from medical records.

#### Compliance to the exercise training program

Exercise frequency (including both supervised and home-based exercise), and the duration of the exercise training period were registered during physiotherapy assessments in the hospital. The number of supervised exercise sessions, Borg-score during resistance exercise, and aerobic exercise intensity were recorded by physiotherapists after each training session, and collected in an exercise record.

#### Measurements of physical fitness and nutritional status

Physical fitness and nutritional status were monitored by a physiotherapist and dietician in the hospital at three timepoints, before (T0) and after neoadjuvant treatment (T1), and after completion of the exercise program just before surgery (T2). Primary outcome measure was the change in exercise capacity during the exercise program (T1-T2). Exercise capacity was measured using the Steep Ramp Test (SRT), a short-term maximal exercise test on a cycle-ergometer, and validated to assess cardiorespiratory fitness (de Backer et al. 2007; Trul-Kreuze et al. 2024). Exercise capacity was quantified by the peak work rate achieved during the SRT, expressed in Watts, which was increased in steps of 25 watts every 10s. Other physical fitness measurements included hand grip strength (HGS) using a Jamar hand-held dynamometer (Peolsson et al. 2001; Bohannon 2019), upper leg muscle function using the Five-Times-Sit-To-Stand Test (FTSTS) (Goldberg et al. 2012; Muñoz-Bermejo et al. 2021), functional mobility using the Timed Up and Go Test (TUG) (Huisman et al. 2014; Podsiadlo and Richardson 1991), fatigue using the Short Fatigue Questionnaire (Alberts et al. 1997), self-reported physical functioning using the Physical Functioning Subscale of the Research and Development-36 (VanderZee et al. 1996), and physical activity level using the Lasa Physical Activity Questionnaire (Stel et al. 2004). Nutritional status was determined in terms of BMI, risk of malnutrition using the Patient-Generated Subjective Global Assessment Short Form (PG-SGA SF) (Jager-Wittenaar and Ottery 2017; Sealy et al. 2018), and nutritional intake, as percentage of the estimated requirements for energy (kcal/day) and protein (g/day) intake. The used formulas and calculations for energy and protein requirements are described in Additional File 1.

### Statistical analysis

Data analyses were performed with the statistical package for the social sciences (SPSS), v29. To describe baseline characteristics, descriptive analyses (mean with SD, and frequencies with percentages) were used. Changes in physical fitness and nutritional status between T0 and T2 were evaluated using linear mixed models with a random intercept, allowing for the inclusion of available data from patients who did not complete all measurements or were lost to follow up at T1 or T2. To explore heterogeneity in preoperative changes in physical fitness and nutritional status among patients, additional analyses were conducted to examine how many patients exhibited a change pattern that corresponds to the average values – defined by a decline in physical fitness and nutritional status from T0 to T1, followed by an improvement from T1 to T2. A decrease from T0 to T1 was defined as a negative value (-), and no change or an improvement as a positive value (+). From T1 to T2, a decrease or no improvement were defined as a negative value (-), and an improvement as a positive value (+). Change patterns in the two periods were combined into four subgroups (--, -+, +-, and ++). Numbers and percentages of patients in the four subgroups were calculated for exercise capacity, HGS, self-reported physical functioning, BMI, and malnutrition risk.

To investigate whether changes in exercise capacity between T0 and T2 differed between patients with a low (≤ 175 Watt) (West et al. 2016; Moran et al. 2016), moderate (176–300 Watt), or high (≥ 300 Watt) exercise capacity at baseline, a linear mixed regression analysis was used with exercise capacity as dependent variable, and time point, exercise capacity at T0, and the interaction term (time point*exercise capacity T0) as independent variables.

To examine whether patient characteristics related to physical fitness, nutritional status, and fatigue differed between responders and non-responders (defined as > 0 Watt or ≤ 0 Watt improvement in exercise capacity during the exercise training program, T1-T2), independent sample T-tests were conducted. Patient characteristics included BMI, exercise capacity, self-reported physical functioning, fatigue, physical activity level, risk of malnutrition and protein intake before the exercise program (T1), as well as changes in these characteristics during neoadjuvant treatment (T0-T1). Variables showing a difference between responders and non-responders (*p* < 0.10), were included in a stepwise, backward multivariable logistic regression analysis, with response to prehabilitation (yes/no) as the dependent variable. Multicollinearity among independent variables were assessed using a correlation matrix (*r* > 0.8).

To evaluate whether the inclusion of patients who ultimately did not undergo surgery (due to ‘wait and see policy’, metastases, frailty or mortality) influenced the study results, physical fitness and nutritional status at T0 were compared between patients who did undergo surgery and patients who did not undergo surgery, using independent sample T-tests. Subsequently, linear mixed model analyses and regression analyses were repeated in a post-hoc analysis restricted to patients who proceeded to surgery following prehabilitation. Statistical significance was set at *p* < 0.05.

## Results

**Fig. 1:**
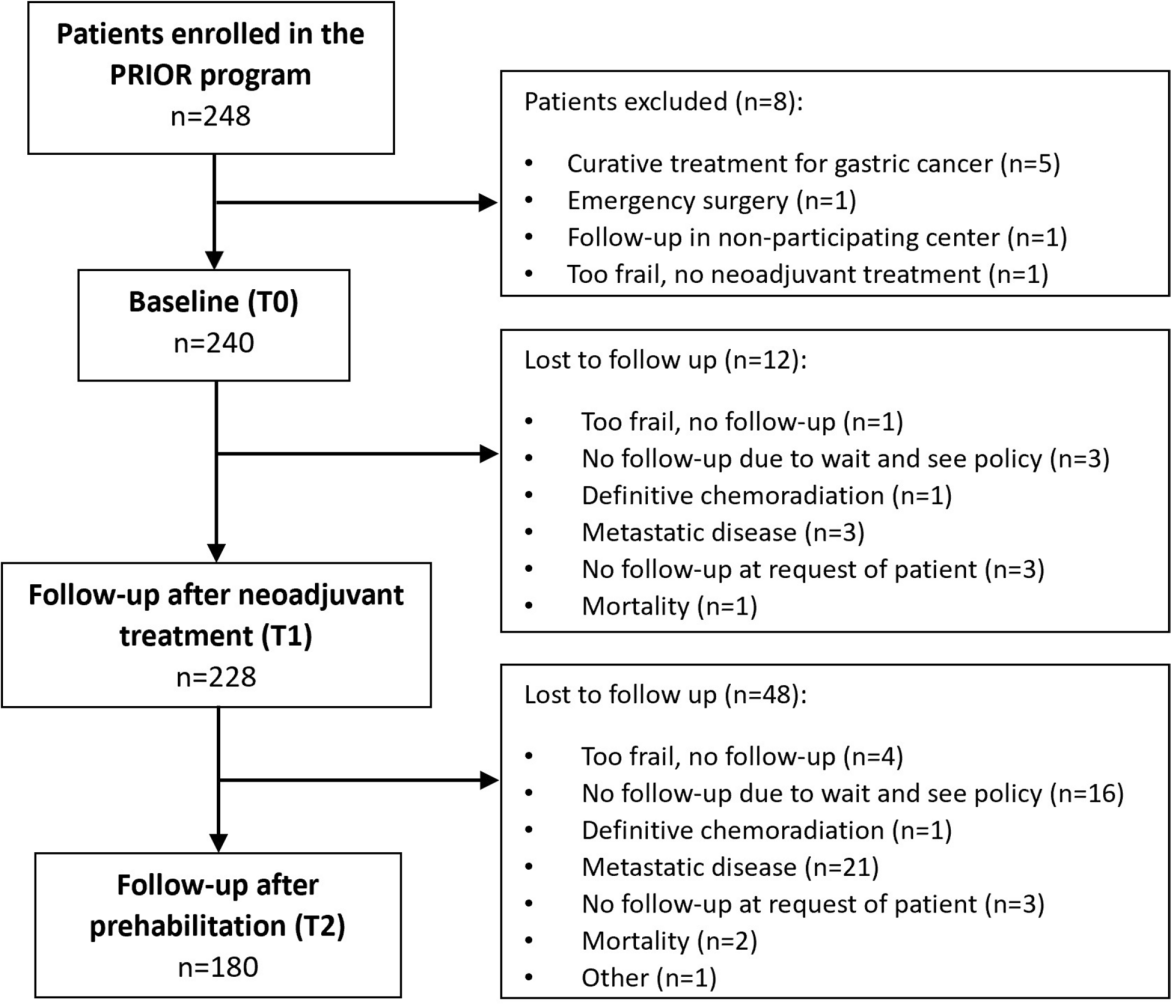
Flowchart of inclusion and follow-up of patients in the PRIOR program. T0: before neoadjuvant treatment; T1: after neoadjuvant treatment; T2: after prehabilitation, before surgery. PRIOR = PReoperative intervention to Improve outcomes in Oesophageal cancer patients after Resection; n = number of patients

In this study, 248 patients with oesophageal cancer enrolled in the PRIOR program. Eight patients were not included, resulting in the inclusion of 240 patients at baseline. In Fig. 1 the reasons for exclusion, and reasons for missing follow-up measurements at T1 and T2 are described. Most common reasons for loss to follow-up were that patients did not undergo surgery due to ‘wait and see policy’ (*n* = 19) or metastases (*n* = 24). Mean age of the included patients was 66.0 (9.3) years and 77.1% were male. Baseline characteristics are described in Table 1.

**Table 1:** Baseline characteristics of participants (*n* = 240)

Characteristics	
Age in years, mean (SD)	66.0 (9.3)
Sex, n (%)	
Male	185 (77.1)
Female	55 (22.9)
Body weight in kg, mean (SD)	81.8 (15.6)
BMI, n (%)	
< 18,5	6 (2.5)
18,5–24,9	93 (38.8)
25–29,9	94 (39.2)
> 30	43 (17.9)
Unknown	4 (1.7)
Co-morbidity, n (%)	
Pulmonary	55 (22.9)
Cardiac	22 (9.2)
Vascular	29 (12.1)
Diabetic	51 (21.3)
Histology, n (%)	
Adenocarcinoma	187 (77.9)
Squamous cell carcinoma	45 (18.8)
Other	8 (3.3)
T-stage, n (%)	
T1	2 (0.8)
T2	56 (23.3)
T3	174 (72.5)
T4	8 (3.3)
N-stage, n (%)	
N0	99 (41.3)
N1	83 (34.6)
N2	48 (20.0)
N3	8 (3.3)
Unknown	2 (0.8)
Tumor location, n (%)	
Cervical – upper third	4 (1.7)
Middle third	24 (10.0)
Lower third	191 (79.6)
Gastro-oesophageal junction	20 (8.3)
Unknown	1 (0.4)
Medical treatment, n (%)	
Chemoradiotherapy and oesophagectomy	175 (72.9)
Chemoradiotherapy, no oesophagectomy	62 (25.8)
Chemotherapy, no oesophagectomy	1 (0.4)
Radiotherapy, no oesophagectomy	2 (0.8)
ASA classification, n (%)	
I	13 (5.4)
II	104 (42.3)
III	71 (29.6)
IV	2 (0.8)
Unknown	50 (20.8)^a^

*SD* Standard deviation, *BMI* Body mass index, *ASA* American Society of Anesthesiologists (ASA)

^a^ASA classification was only registered in patients who underwent surgery

### Compliance to exercise training program

Of the 228 patients included in the study at T1, 202 (88.6%) patients were referred to a physiotherapist to follow the supervised exercise program. Twenty-six patients did not engage in the supervised exercise program: 17 patients (7.5%) declined to participate, or preferred a program without physiotherapeutic supervision, and 9 patients (3.9%) were not referred due to missing the physiotherapeutic assessment at T1. These 26 patients were retained in the analyses. The median exercise training period from T1 to T2 was 9.3 weeks (IQR: 6.3–12.4). With inclusion of home-based exercise sessions, 12 patients (6.7%) performed less than two exercise sessions per week, 62 patients (34.4%) performed two sessions per week, and 75 patients (41.7%) three or more than three sessions per week. From 31 patients (17.2%) exercise frequency was unknown. Based on exercise records from 107 patients, the median number of supervised exercise sessions was 12 (IQR: 8–16). Resistance exercise was performed at an average Borg-score (0–10) of 5.0 (SD 1.3). Intensity of aerobic exercise was 36.5 (SD 16.5)% of the peak workload (T1) during the first exercise session, and 42.5 (SD 12.8)% of peak workload (T1) during the last exercise session.

### Changes in physical fitness and nutritional status during chemoradiotherapy (T0-T1) and exercise training program (T1-T2)

In Table 2 the physical and nutritional measurements at T0, T1 and T2 are presented. Between T0 and T1 physical fitness showed a significant deterioration in terms of HGS (−1.2 [−2.1; −0.3] kg; *p* < 0.01), exercise capacity (−24 [−31; −18] watt; *p* < 0.001), fatigue (+ 5.2 [4.1; 6.4]; *p* < 0.001), self-reported physical functioning (−11.2 [−14.0; −8.5] points; *p* < 0.001), and physical activity level (−160 [−242; −78] METmin/day; *p* < 0.001)). No significant changes were observed in FTSTS and TUG performance during T0-T1 (*p* = 0.61 and 0.19, respectively). Between T1 and T2, all physical fitness measures showed significant improvement (*p* < 0.01). Compared to T0, values at T2 for exercise capacity (+ 8 [1;16] watt; *p* = 0.02), FTSTS (−1.3 [−1.9; −0.8] s; *p* < 0.001), and fatigue (−1.8 [−3.0; −0.6]; *p* = 0.001) were significantly improved (*p* < 0.03).

**Table 2:** Changes in physical fitness and nutritional parameters during the preoperative period

Measurements	n	T0	n	T1	ΔT0-T1	*p*-value	n	T2	ΔT1-T2	*p*-value	ΔT0-T2	*p*-value
Body mass index (kg/m^2^)	236	26.3^a^ (25.7; 26.8)	222	26.0 (25.4; 26.5)	−0.3 (−0.6; −0.0)	.02	182	26.5 (25.9; 27.0)	+ 0.5 (0.2; 0.8)	<.001	+ 0.2 (−0.1; 0.5)	.25
Energy intake (% of estimated requirement)	223	86.7 (83.9; 89.6)	202	86.1 (83.1; 89.1)	−0.6 (−4.9; 3.7)	>.99	114	96.1 (92.2; 100.0)	+ 10.0 (4.7; 15.3)	<.001	+ 9.4 (4.1; 14.6)	<.001
Protein intake (% of estimated requirement)	222	70.3 (67.3; 73.3)	203	80.9 (77.8; 84.1)	+ 10.6 (6.0; 15.3)	<.001	115	82.7 (78.7; 86.8)	+ 1.8 (−3.9; 7.5)	>.99	+ 12.4 (6.8; 18.0)	<.001
PG-SGA Short Form^b^	197	6.1 (5.4; 6.7)	175	6.2 (5.5; 6.9)	+ 0.1 (−0.9; 1.1)	>.99	109	2.6 (1.8; 3.4)	−3.6 (−4.8; −2.4)	<.001	−3.5 (−4.7; −2.3)	<.001
Exercise capacity (peak workload SRT, watt)	234	233 (224; 242)	195	208 (199; 218)	−24 (−31; −18)	<.001	142	241 (232; 251)	+ 33 (25; 40)	<.001	+ 8 (1; 16)	.02
Exercise capacity (peak workload SRT, watt/kg)	233	2.87 (2.77; 2.98)	194	2.61 (2.50; 2.72)	−0.26 (−0.33;−0.18)	<.001	139	2.96 (2.85; 3.08)	+ 0.35 (0.27; 0.44)	<.001	+ 0.09 (0.01; 0.18)	.03
Hand grip strength (kg)	236	39.7 (38.5; 41.0)	206	38.5 (37.0; 40.0)	−1.2 (−2.1; −0.3)	<.01	145	40.1 (38.5; 41.6)	+ 1.6 (0.5; 2.6)	.001	+ 0.4 (−0.7; 1.4)	>.99
Five Times Sit to Stand Test (s)	221	10.8 (10.3; 11.3)	191	10.5 (10.0; 11.1)	−0.3 (−0.8; 0.2)	.61	137	9.5 (8.9; 10.0)	−1.1 (−1.6; −0.5)	<.001	−1.3 (−1.9; −0.8)	<.001
Timed Up and Go Test (s)	233	6.6 (6.2; 7.0)	200	6.9 (6.5; 7.4)	+ 0.3 (−0.1; 0.8)	.19	146	6.3 (5.8; 6.8)	−0.6 (−1.1; −0.2)	<.01	−0.3 (−0.8; 0.2)	.36
Fatigue^c^	227	10.5 (9.7; 11.4)	192	15.8 (14.9; 16.7)	+ 5.2 (4.1; 6.4)	<.001	150	8.7 (7.8; 9.7)	−7.1 (−8.3; −5.8)	<.001	−1.8 (−3.0; −0.6)	.001
Self-reported physical functioning^d^	228	85.8 (83.3; 88.2)	196	74.5 (71.9; 77.1)	−11.2 (−14.0; −8.5)	<.001	150	86.7 (83.9; 89.5)	+ 12.2 (9.1; 15.3)	<.001	+ 0.9 (−2.1; 4.0)	>.99
Physical activity level (METmin/day)	210	495 (443; 548)	181	335 (279; 392)	−160 (−242; −78)	<.001	142	561 (498; 624)	+ 226 (135; 316)	<.001	+ 66 (−23; 154)	.22

*PG-SGA* Patient-Generated Subjective Global Assessment, *SRT* Steep Ramp Test, *MET* Metabolic Equivalent Task, *T0* Before neoadjuvant treatment,*T1* After neoadjuvant treatment, *T2* After prehabilitation, immediately before surgery

^a^Values are displayed as means (95% CI); Bonferroni correction is performed to correct for multiple comparisons

^b^Higher values are associated with a higher risk of malnutrition

^c^Scoring fatigue: 4 = no fatigue, 28 = maximum fatigue

^d^Scoring self-reported physical functioning: 0 = severe limitations, 100 = no limitations

In terms of nutritional status, mean BMI significantly decreased between T0 and T1 (−0.3[−0.6; −0.0] kg/m^2^; *p* = 0.02), while energy intake and malnutrition risk remained stable (*p* > 0.99), and protein intake increased (+ 10.6 [6.0; 15.3]%; *p* < 0.001). From T1 to T2, BMI (+ 0.5 [0.2; 0.8] kg/m^2^; *p* < 0.001), energy intake (+ 10.0 [4.7; 15.3]%; *p* < 0.001), and malnutrition risk (−3.6 [−4.8; −2.4]; *p* < 0.001) showed significant improvement, whereas protein intake remained stable (*p* > 0.99). Compared to baseline (T0), BMI at T2 did not differ significantly (*p* = 0.25), but energy intake (+ 9.4 [4.1; 14.6]%; *p* < 0.001), protein intake (+ 12.4 [6.8; 18.0]%; *p* < 0.001), and malnutrition risk (−3.5 [−4.7; −2.3]; *p* < 0.001) were all significantly improved at T2.

The percentage of patients who followed a change pattern that corresponds to the average values (defined as a decline from T0 to T1, followed by an improvement from T1 to T2 – pattern -+), ranged from 36.2 to 57.8%, depending on the outcome measure (Table 3). Other patients exhibited alternative trajectories in preoperative changes, including 3.5 to 16.7% of patients following ‘pattern --’, 20.6 to 28.3% showing ‘pattern +-‘ and 8.9 to 29.1% following ‘pattern + + ’.

**Table 3:** Patterns of change in physical fitness and nutritional status in the preoperative period

Measurements	Pattern of change in physical fitness and nutritional status during neoadjuvant treatment (T0-T1) and exercise training program (T1-T2)^a^			
	**- -**	**- + **	** + -**	** + + **
Exercise capacity, n (%)	20 (15.3)	65 (49.6)	27 (20.6)	19 (14.5)
Hand grip strength, n (%)	23 (16.7)	50 (36.2)	39 (28.3)	26 (18.8)
Self-reported physical functioning, n (%)	13 (9.6)	78 (57.8)	32 (23.7)	12 (8.9)
Body Mass Index, n (%)	20 (14.7)	57 (41.9)	30 (22.1)	29 (21.3)
Malnutrition risk, n (%)^b^	3 (3.5)	36 (41.9)	22 (25.6)	25 (29.1)

^a^Patterns of change in physical fitness or nutritional status

^b^Missing values in subscales of the Patient-Generated Subjective Global Assessment short form resulted in a lower number of patients, compared to other outcome measures

**-- **decrease during neoadjuvant treatment (T0-T1) and decrease or no change during exercise program (T1-T2)

**- + **decrease during neoadjuvant treatment (T0-T1) and improvement during exercise program (T1-T2)

** +- **improvement or no change during neoadjuvant treatment (T0-T1) and decrease or no change during exercise program (T1-T2)

** + + **improvement or no change during neoadjuvant treatment (T0-T1) and improvement during exercise program (T1-T2)

### Impact of baseline level on the preoperative course of exercise capacity

**Fig. 2:**
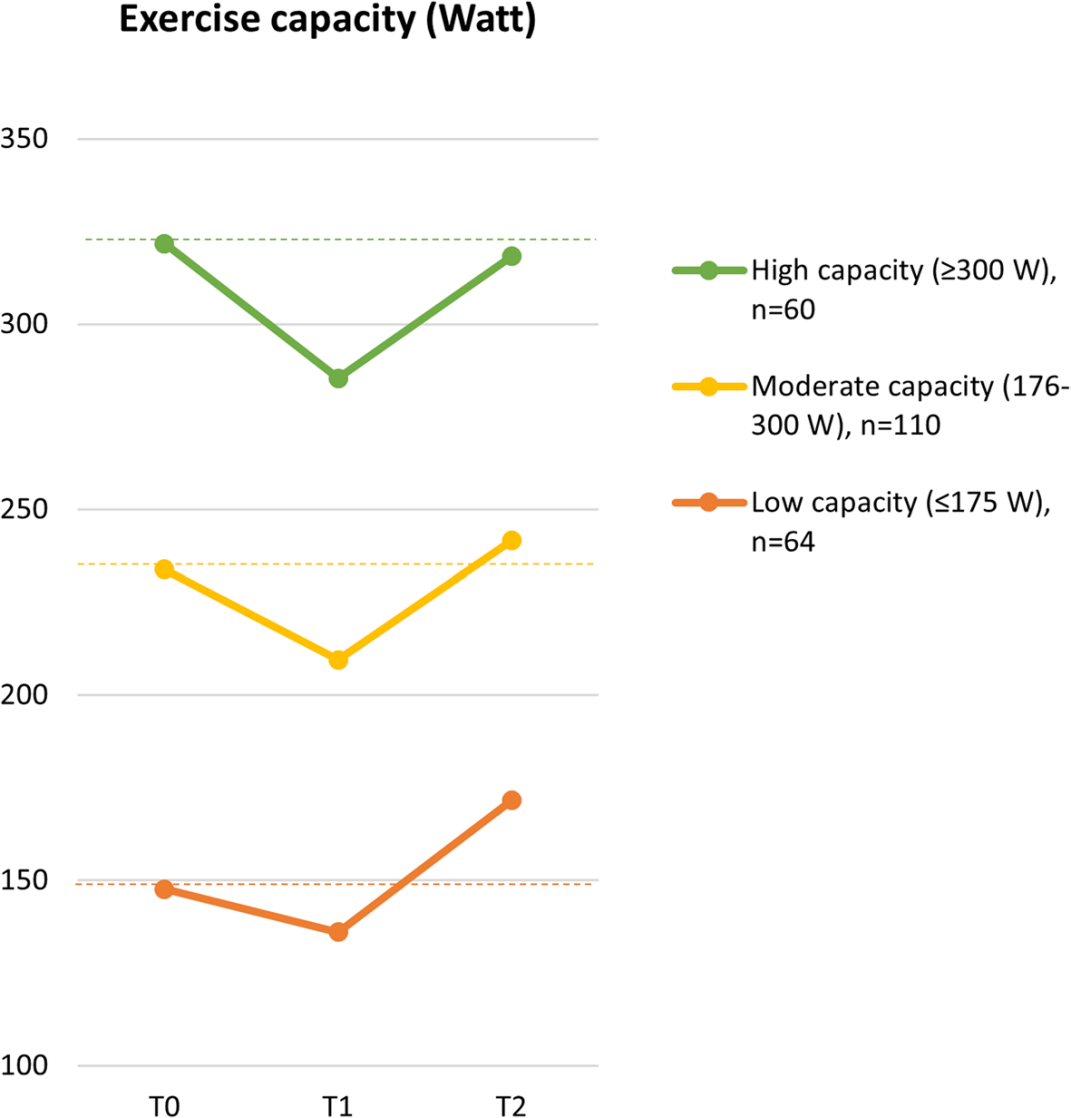
Preoperative changes in exercise capacity for patients with low, moderate and high baseline exercise capacity. T0: before neoadjuvant treatment; T1: after neoadjuvant treatment; T2: after prehabilitation, before surgery

On average, exercise capacity deteriorated from T0 to T1 by −24.2 [−31.7; −17.7] watts, followed by an improvement from T1 to T2 of + 32.7 [25.2; 40.2] watts. Figure 2 illustrates the changes in exercise capacity (in watts), stratified by baseline level (T0): low (≤ 175 watts), moderate (176–300 watts), and high (≥ 300 watts) exercise capacity. Patients with low baseline exercise capacity (*n* = 64) showed a smaller decline from T0 to T1 (−11.4 [−21.9; −0.9] watts) compared to patients with moderate (−24.5 [−32.0; −17.1] watts; *n* = 110) and high (−36.2 [−46.0; −26.5] watts; *n* = 60) baseline capacity, with *p*-values of 0.048 and < 0.001, respectively. Improvements from T1 to T2 did not significantly differ across the three groups: low (+ 35.3 [21.7; 48.9] watts), moderate (+ 32.0 [23.8; 40.3] watts), and high (+ 32.9 [21.8; 44.0] watts) baseline capacity, with *p*-values ranging from 0.69 to 0.90.

### Differences in physical fitness level, nutritional status and fatigue between responders and non-responders to the exercise program

Exercise capacity was assessed at both T1 and T2 in 133 patients. Eighty-six (64.7%) patients showed an improvement in exercise capacity (> 0 Watt) from T1 to T2 and were classified as responders, while 47 (35.3%) patients did not improve and were classified as non-responders. Table 4 presents the differences in physical fitness, nutritional status, and fatigue between responders and non-responders. Responders demonstrated a greater decline in exercise capacity during neoadjuvant treatment (T0-T1) (*p* < 0.001), a lower exercise capacity at T1 (*p* = 0.001), lower self-reported physical functioning (*p* = 0.01), and higher levels of fatigue at T1 (*p* = 0.01), compared to non-responders. Multivariable analysis demonstrated that a greater decline in exercise capacity during neoadjuvant treatment (Odds Ratio 0.97 (0.96; 0.99); *p* < 0.001), and a lower exercise capacity at T1 (Odds Ratio 0.99 (0.99; 1.00); *p* = 0.058) remained as independent variables in the model for response to the exercise program.

**Table 4:** Physical fitness and nutritional status of responders and non-responders to the exercise program, *n* = 133

	Responders (***n*** = 86)^a^ mean (SD)	Non-responders (***n*** = 47) mean (SD)	***P***-value
Δ Body Mass Index T0-T1	−0.55 (1.47)	−0.17 (1.24)	.14
Body Mass Index T1	26.3 ± 4.2	25.8 (4.9)	.57
Δ Exercise capacity T0-T1 (Watt)	−35.4 (37.5)	−6.7 (27.4)	<.001
Exercise capacity T1 (Watt)	206.7 (68.5)	246.8 (62.5)	.001
Δ Self-reported physical functioning (0–100%) T0-T1	−12.2 (16.4)	−8.8 (12.4)	.23
Self-reported physical functioning (0–100%) T1	73.8 (20.3)	83.1 (17.2)	.01
Δ Fatigue T0-T1 (4–28)	+ 6.5 (6.7)	+ 5.4 (5.9)	.37
Fatigue T1 (4–28)	16.8 (6.7)	13.4 (6.0)	.01
Δ Physical activity T0-T1 (METmin/day)	−175 (398)	−184 (372)	.91
Physical activity T1 (METmin/day)	305 (289)	398 (304)	.10
Δ PG-SGA SF T0-T1	+ 0.6 (6.7)	+ 0.3 (4.4)	.82
PG-SGA SF T1	6.4 (5.6)	4.8 (4.3)	.14
Δ Protein intake T0-T1 (%)	+ 10.2 (28.1)	+ 4.2 (20.9)	.23
Protein intake T1 (%)	79.5 (25.6)	81.7 (19.1)	.58

*MET* Metabolic Equivalent Task, *PG-SGA SF* Patient Generated Subjective Global Assessment short form (malnutrition risk), *T0* Before neoadjuvant treatment, *T1* After neoadjuvant treatment

^a^Responders are defined as > 0 Watt improvement of exercise capacity during the exercise program (T1-T2) and non-responders as ≤ 0 Watt improvement during the exercise program (T1-T2)

### Post-hoc analyses on patients who underwent surgery

Post-hoc analyses showed that patients who eventually did not undergo surgery had a significantly lower energy intake (81.7% versus 88.6%, *p* = 0.04), a higher risk of malnutrition (7.2 versus 5.7, *p* = 0.04), a lower exercise capacity (214 Watt versus 240 Watt, *p* = 0.01), and a higher degree of fatigue (12.0 versus 9.9, *p* = 0.04) at T0 compared to patients who did undergo surgery (Additional File 2, Table A1). The results of the post-hoc analyses, with exclusion of non-surgical patients (Additional File 2, Figure A1), showed that the change in physical fitness and nutritional status from T0 to T1 and from T1 to T2 remained similar to the initial analyses including also non-surgical patients. The impact of baseline level, and differences between responders and non-responders also showed similar results compared to the initial analyses (Additional File 2, Figure A2, Table A2 and Table A3).

## Discussion

In this study, we described preoperative changes in physical fitness and nutritional status among patients with oesophageal cancer who participated in a prehabilitation program consisting of an exercise training program and nutritional support, and explored which factors were associated with the response to prehabilitation. On average, patients demonstrated improvements in both physical fitness and nutritional status during prehabilitation, recovering from the impact of neoadjuvant treatment to baseline values or higher. Notably, patients with low baseline fitness were also able to improve their fitness during prehabilitation. However, substantial heterogeneity was observed in preoperative changes in physical fitness and nutritional status across individual patients, underscoring the importance of regular monitoring and the need for personalized interventions.

Our findings demonstrate that patients are indeed able to recover from the impact of chemoradiotherapy during a prehabilitation program, consistent with previous studies on prehabilitation in patients with oesophageal cancer (Halliday et al. 2021b; Argudo et al. 2021). In contrast, studies involving patients who did not participate prehabilitation, have reported significant declines in physical fitness 4 to 6 weeks after completing neoadjuvant treatment (Guinan et al. 2017; Döbeln et al. 2016; Thomson et al. 2018; Reijneveld et al., 2022). These results support the potential of prehabilitation to mitigate the negative effects of neoadjuvant treatment. Nonetheless, our findings indicate considerable heterogeneity in preoperative changes in physical fitness and nutritional status across individual patients. Approximately half of the patients followed a change pattern that corresponds to the average values, defined as a deterioration during neoadjuvant treatment (T0 -T1), followed by an improvement during the exercise training program (T1-T2). However, a substantial proportion of patients either maintained or improved their physical fitness and nutritional status during T0-T1, or did not show improvement during T1-T2. With regard to exercise capacity, which is an important predictor of postoperative recovery (Sheill et al. 2020), about one-third of patients did not show improvement during the preoperative exercise program. Further analyses revealed that non-responders generally had higher physical fitness prior to starting prehabilitation, experienced less decline in physical fitness during neoadjuvant treatment, and reported lower levels of fatigue compared to responders. Our finding that patients with a lower physical fitness level show more improvement during prehabilitation is in agreement with previous studies demonstrating more improvement in less fit patients with colorectal and oesophageal cancer (Halliday et al. 2021b; Minnella et al. 2016; Cate et al. 2024). Moreover, in our study, a greater decline in fitness during neoadjuvant treatment was independently associated with being a responder, suggesting that patients who experience substantial deterioration during chemoradiotherapy, are still capable of regaining fitness through targeted exercise. Additionally, higher fatigue levels were associated with a greater likelihood of responding to prehabilitation, possibly due to lower baseline physical activity levels in patients with a high level of fatigue, and greater potential for improvement once exercise is initiated (Reijneveld et al. 2024).

In terms of nutritional status, our study found an increase in both protein and energy intake during the preoperative period, with BMI values returning to baseline levels following prehabilitation. During neoadjuvant treatment, the increase in protein intake was particularly notable, while energy intake remained stable in this period. This rise in protein intake is most likely attributable to the nutritional counseling, initiated before or during neoadjuvant treatment, which emphasized the use of protein-enriched food. During the subsequent period of exercise training, energy intake increased considerably, which may be partly attributed to an improvement in dysphagia, and especially the ability to eat solid food after finishing neoadjuvant treatment (Sunde et al. 2019). Nutritional counseling during the preoperative period appears to have contributed to the restoration of BMI to baseline values, contrasting with previous studies that reported continued preoperative weight loss up to the time of surgery (Movahed et al. 2020; Ericson et al. 2020). Although average BMI increased during prehabilitation in our study, it remains unclear whether this weight gain also reflected an increase in muscle mass, as body composition was not assessed as part of stadard care. In oncological patients, there is a high risk of muscle loss and sarcopenia due to elevated metabolic demands caused by the cancer itself and side effects of neoadjuvant treatment (Gortan Cappellari et al. 2022). Notably, a substantial proportion of patients in our study were already overweight (39%) or obese (18%) at baseline, placing them at risk of sarcopenic obesity—a condition associated with poorer postoperative outcomes (Gortan Cappellari et al. 2022). Therefore, routine assessment of body composition is strongly recommended to identify patients at risk of sarcopenia and to evaluate the effectiveness of prehabilitation interventions.

A question arising from clinical practice was whether patients with low baseline physical fitness level are able to improve their fitness during prehabilitation. To address this, we analyzed the preoperative trajectory of exercise capacity separately for patients with low, moderate, and high baseline exercise capacity. Based on the exploratory findings of this study, patients with low exercise capacity responded to the exercise training program to a similar extent as patients in the other subgroups. Notably, because the decline in fitness during neoadjuvant treatment was relatively small in patients with a low capacity, their exercise capacity improved to levels above baseline after prehabilitation. Despite the observed improvement from 136 to 172 watts, patients in this group still demonstrated substantially lower exercise capacity compared to patients with moderate or high baseline capacity. Since a low preoperative fitness level is associated with an increased risk of postoperative complications and overall morbidity (Sheill et al. 2020; Bor et al. 2021), optimizing preoperative fitness is crucial to reduce a patients’ risk profile. Patients with poor baseline fitness may benefit from a longer and more intensive preoperative training period to enhance their physical fitness level. Therefore, it is critical that patients with low fitness levels make optimal use of the available preoperative window to enhance their chances of a good postoperative recovery.

This study demonstrates that a personalized prehabilitation program, integrated into the standard curative pathway, enhances preoperative physical fitness and nutritional status in patients with oesophageal cancer. The large and representative group of patients followed a personalized program, adapted to individual capacity and preferences, thereby supporting the generalizability of the findings to patients awaiting oncological surgery. Logistically, monitoring and referring patients for prehabilitation is quite challenging. Nevertheless, only 4% of patients were not referred for the exercise training program due to logistic issues, indicating that the referral of patients for the exercise program was successful. Furthermore, 76% of the patients participated in the exercise program at least twice weekly, indicating that the majority of patients was compliant to the intervention. It should be noted, however, that the average intensity of aerobic exercise retained below the recommended intensity of 40–50% of SRT peak workload, with only a minimal increase from 36.5% to 42.5% over the exercise period. Patients may have been unable to attain the recommended exercise intensity due to adverse effects associated with neoadjuvant treatment—such as fatigue, nausea, and weight loss—which could have compromised both their physical ability and motivation to adhere to the prescribed exercise intensity. Moreover, physiotherapists supervising the program may have been cautious to increase the exercise intensity in this specific patient population. Given the limited timeframe before surgery, achieving an optimal exercise intensity is of critical importance to avoid both overtraining and undertraining. Therefore, regular monitoring of physical fitness throughout the exercise program is essential to enable timely adjustments. Enhanced expertise in exercise intensity during prehabilitation could support the development of more precisely tailored exercise programs.

The use of data derived from standard care contributed to a high inclusion rate of patients and a representative study population, which is a strength of this study. However, certain limitations must also be acknowledged. Logistical challenges in scheduling assessments as part of standard care led to instances of missing data. Regarding compliance to the exercise program, the number of home-based exercise sessions was based on patient self-report, which may be subject to recall bias or overestimation. Moreover, exercise frequency and intensity during the exercise training program were not always documented, as complete exercise reports were missing for some patients. Furthermore, in this study, we chose to include all patients who initiated a curative treatment trajectory, including patients who ultimately did not undergo surgery due to metastases or a ‘wait & see policy’. While this subgroup reflects the clinical reality of the oesophageal cancer population, their lower physical fitness levels compared to patients who completed the full curative pathway may have introduced bias. To address this, we conducted post-hoc analyses excluding non-surgical patients, which yielded results consistent with the initial findings.

In conclusion, physical fitness and nutritional status improved during a prehabilitation program consisting of an exercise training program and nutritional support in patients with oesophageal cancer. On average, patients recovered from the impact of neoadjuvant treatment, reaching baseline levels or above. Nevertheless substantial variability in preoperative changes across individuals highlights the importance of regular monitoring and personalized interventions. Our findings indicate that lower physical fitness and higher fatigue levels before exercise training, and greater decline in fitness during neoadjuvant treatment, were associated with a positive response to prehabilitation. Improvements in physical fitness were comparable across patients with low, moderate, and high exercise capacity at diagnosis, indicating that even patients with initially low fitness can effectively enhance their preoperative fitness level through personalized prehabilitation.

## Supplementary Information

Supplementary Material 1. 

## Data Availability

The dataset used during the current study is available from the corresponding author, EAER, upon reasonable request.

## Abbreviations

PRIOR: PReoperative Intervention to improve outcomes in patients with oesophageal cancer after resection
BMI: Body Mass Index
ASA: American Society of Anesthesiologists
PG-SGA SF: Patient-generated subjective global assessment short form
HGS: Hand grip strength
FTSTS: Five times sit to stand test
TUG: Timed up and go test
IQR: Interquartile range
