# Supplementary material for: Response to a prehabilitation program for patients with oesophageal cancer: an observational study
Source: Perioper Med (Lond). 2025 Dec 19;14:142. doi: 10.1186/s13741-025-00633-6 (PMC12752240; doi:10.1186/s13741-025-00633-6)
Supplement: Supplementary file 1 — Supplementary Material 1. [file 13741_2025_633_MOESM1_ESM.pdf]

Additional File 1:

Used formulas for calculating the nutritional intake

Nutritional intake was recorded in terms of protein and energy intake, expressed as a percentage of the calculated requirements. Dieticians performed a 24-hour recall and registered the protein and energy intake from the past day, including the amount of oral nutritional supplements and tube feeding [1]. Protein and energy requirements were calculated based on the European Society for Clinical Nutrition and Metabolism (ESPEN) guidelines for patients with cancer and the Dutch dietary guidelines [2,3]. Protein requirements for patients with a BMI up to 25 were calculated as 1.5 g protein/kg body weight per day [4]. For patients with a BMI <20 kg/m2, body weight was corrected to a BMI of 20 kg/m2 to prevent underestimation of protein requirements [5]. For patients with a BMI >25 kg/m2, protein requirements were calculated as 1.9 g protein/kg lean body mass. Lean body mass was estimated using the formula of Gallagher [6]. Requirements for resting energy expenditure were calculated using the world health organization (WHO) or Harris and Benedict formulas [3,7]. For patients with a BMI <30 kg/m2, the WHO formula was used and for patients with a BMI ≥30 kg/m2 the formula from Harris & Benedict. Dieticians used additional percentages for metabolic stress and physical activity level up to a maximum of 50% to estimate the total energy expenditure [7,8].

References

[1] Jonnalagadda SS, Mitchell DC, Smiciklas-Wright H, Meaker KB, Van Heel N, Karmally W, Ershow AG, Kris-Etherton PM. Accuracy of energy intake data estimated by a multiple-pass, 24-hour dietary recall technique. J Am Diet Assoc 2000 Mar;100(3):303–11.

[2] Muscaritoli M, Arends J, Bachmann P, Baracos V, Barthelemy N, Bertz H, Bozzetti F, Hütterer E, Isenring E, Kaasa S, Krznaric Z, Laird B, Larsson M, Laviano A, Mühlebach S, Oldervoll L, Ravasco P, Solheim TS, Strasser F, de van der Schueren M, Preiser J, Bischoff SC. ESPEN practical guideline: Clinical nutrition in cancer. Clin Nutr 2021 May;40(5):2898–913.

[3] Dutch malnutrition guideline [Internet]; c2019 [cited 2024 July 25]. Available from: https://www.kenniscentrumondervoeding.nl/wp-content/uploads/2022/04/SoV01-Richtlijn-Ondervoeding-februari-2019-met-addendum-september-2021.pdf.

[4] Weijs PJM, Sauerwein HP, Kondrup J. Protein recommendations in the ICU: G protein/kg body weight - which body weight for underweight and obese patients? Clin Nutr 2012 Oct;31(5):774–5.

[5] Velzeboer L, Huijboom M, Weijs P, Engberink M, Kruizenga H. How to calculate the protein needs in under- and overweight persons?
does gallagher's formula give a better estimation? Ned Tijdschr Voeding Diëtetiek 2017;72(1):1.

[6] Gallagher D, Heymsfield SB, Moonseong H, Jebb SA, Murgatroyd PR, Sakamoto Y. Healthy percentage body fat ranges: An approach for developing
guidelines based on body mass index. 2000;72:694–701.

[7] Kruizenga HM, Hofsteenge GH, Weijs PJM. Predicting resting energy expenditure in underweight, normal weight, overweight, and obese adult hospital patients. Nutr Metab (Lond) 2016 Nov 24;13:85,3. eCollection 2016.

[8] Elia M. Insights into energy requirements in disease. Public Health Nutr 2005 Oct;8(7A):1037–52.

Additional File 2:

Post-hoc analyses on patients who did underwent oesophagectomy:

Table A1: Baseline physical fitness and nutritional status in patients who underwent surgery and in patients who did not undergo curative surgery.

**Baseline level (T0)**	**Patients who underwent oesophagectomy (n=175)**	**Patients who did not undergo oesophagectomy (n=65)**	**P-value**
Body Mass Index (kg/m^2)	26.4 ± 4.2	25.8 ± 3.9	.357
Energy intake (% of estimated requirement)	88.6 ± 23.2	81.7 ± 18.9	.039*
Protein intake (% of estimated requirement)	71.6 ± 19.7	67.3 ± 20.4	.080
PG-SGA Short Form#	5.7 ± 4.3	7.2 ± 4.8	.037*
Exercise capacity (peak workload SRT, watt)	240 ± 69	214 ± 73	.012*
Exercise capacity (peak workload SRT, watt/kg)	2.94 ± 0.83	2.70 ± 0.81	.049*
Hand grip strength (kg)	39.8 ± 10.4	38.6 ± 11.7	.435
Five Times Sit to Stand Test (s)	10,6 ± 4.0	11.4 ± 3.6	.166
Timed Up and Go Test (s)	6,5 ± 3.1	6.7 ± 1.6	.553
Fatigue<br>*Scoring: 4=no fatigue, 28=maximum fatigue*	9.9 ± 6.2	12.0 ± 7.1	.043*
Self-reported physical functioning<br>*Scoring: 0=severe limitations, 100=no limitations*	86.1 ± 18.0	84.7 ± 18.9	.591
Physical activity level (METmin/day)	524 ± 395	426 ± 388	.102

PG-SGA: Patient-Generated Subjective Global Assessment; SRT: Steep Ramp Test; MET: Metabolic Equivalent Task

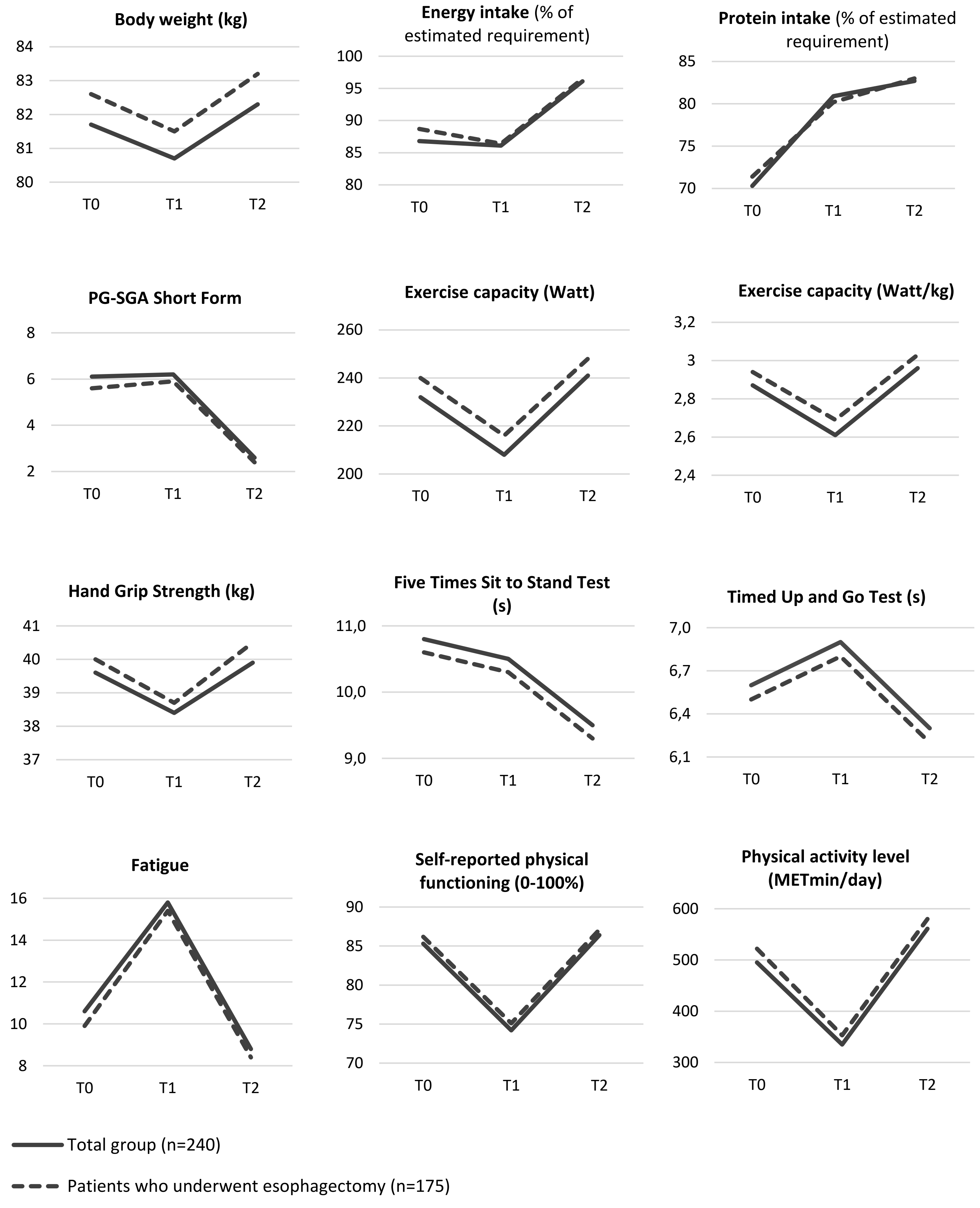

Figure A1: Changes in physical fitness and nutritional parameters during the preoperative period in all patients (solid line, n=240) and in patients who underwent oesophagectomy (dotted line, n=175).

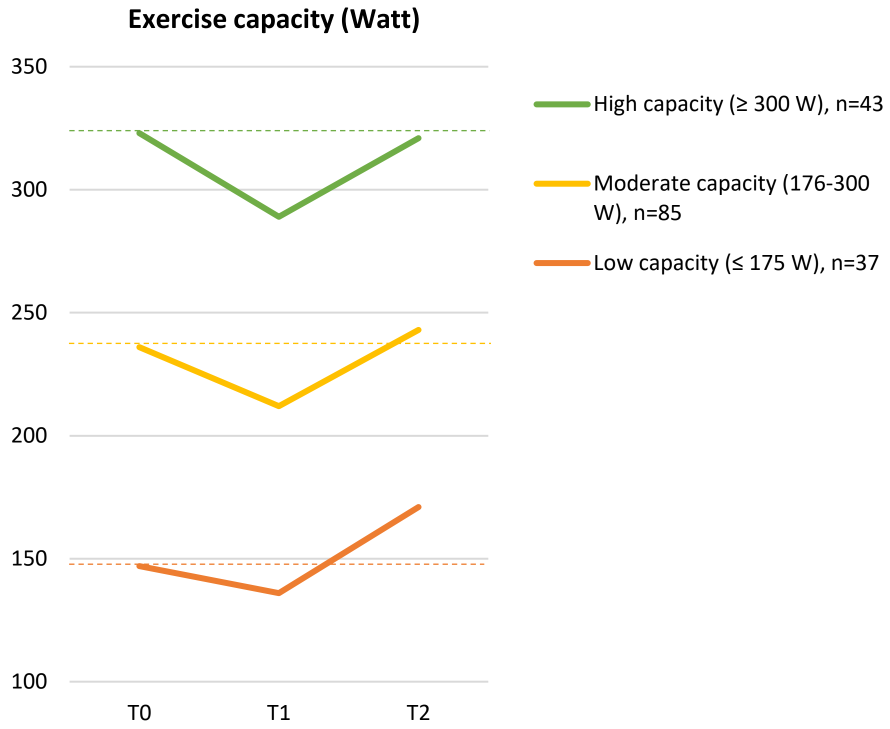

Figure A2: Preoperative changes in exercise capacity for patients with low (≤175 Watt), moderate (176-300 Watt), and high (≥300 Watt) baseline exercise capacity
T0: before neoadjuvant treatment; T1: after neoadjuvant treatment; T2: after prehabilitation, before surgery.

During neoadjuvant treatment (T0-T1), patients with a low capacity at baseline showed a significant less decrease in exercise capacity compared to patients with a high capacity at baseline (p=.009). Differences between the low and moderate capacity group, and between the moderate and high capacity group were not statistically significant (p=.091 and p=.170). The response to prehabilitation (T1-T2) was similar in all three groups (p ranging from .909).

Table A2: Physical fitness and nutritional status of surgical patients who did respond and who did not respond to prehabilitation, n=123

Mean change in exercise capacity: 32,7 ± 39,6 Watt

	**Responders (n=78)*** mean ± SD	**Non-responders (n=45)** mean ± SD	**P-value**
Δ Body Mass Index T0-T1	-0.59 ± 1.49	-0.15 ± 1.27	.107
Body Mass Index T1	26.3 ± 4.3	25.7 ± 4.9	.525
Δ Exercise capacity T0-T1 (Watt)	-35.4 ± 38.5	-7.0 ± 27.4	<.001
Exercise capacity T1 (Watt)	203.2 ± 68.3	245.6 ± 63.6	<.001
Δ Self-reported physical functioning (0-100%) T0-T1	-12.5 ± 16.8	-8.0 ± 11.9	.130
Self-reported physical functioning (0-100%) T1	72.7 ± 19.2	83.6 ± 17.4	.003
Δ Fatigue T0-T1 (4-28)	+6.6 ± 6.8	+5.2 ± 5.9	.277
Fatigue T1 (4-28)	17.0 ± 6.6	13.1 ± 6.0	.002
Δ Physical activity T0-T1 (METmin/day)	-176 ± 410	-188 ± 376	.884
Physical activity T1 (METmin/day)	309 ± 290	404 ± 306	.101
Δ PG-SGA SF T0-T1	+0.8 ± 6.8	+0.4 ± 4.5	.770
PG-SGA SF T1	6.5 ± 5.8	4.7 ± 4.0	.081
Δ Protein intake T0-T1 (%)	+11.1 ± 28.4	+3.6 ± 20.9	.146
Protein intake T1 (%)	80.1 ± 25.8	81.0 ± 18.8	.843

MET: Metabolic Equivalent Task; T0: before neoadjuvant treatment; T1: after neoadjuvant treatment; PG-SGA SF: Patient Generated Subjective Global Assessment short form (malnutrition risk).

*Responders are defined as >0 Watt improvement of exercise capacity during prehabilitation (T1-T2) and non-responders as ≤0 Watt improvement during prehabilitation (T1-T2).

Table A3: Multivariable logistic regression analysis on the response to prehabilitation (> or ≤0 Watt improvement).

**R^2 = .226**	**Odds Ratio (95% CI)**	**P-value**
Δ Exercise capacity T0-T1 (Watt)	0.99 (0.97; 1.00)	.049
Exercise capacity T1 (Watt)	0.99 (0.98; 1.00)	.007
